# Frequency of Boiled Potato Consumption and All-Cause and Cardiovascular Disease Mortality in the Prospective Population-Based HUNT Study

**DOI:** 10.3389/fnut.2021.681365

**Published:** 2021-07-19

**Authors:** Trine Moholdt, Tom I. L. Nilsen

**Affiliations:** ^1^Department of Circulation and Medical Imaging, Norwegian University of Science and Technology, Trondheim, Norway; ^2^Women's Clinic, St Olavs Hospital, Trondheim University Hospital, Trondheim, Norway; ^3^Department of Public Health and Nursing, Norwegian University of Science and Technology, Trondheim, Norway; ^4^Clinic of Anaesthesia and Intensive Care, St Olavs Hospital, Trondheim University Hospital, Trondheim, Norway

**Keywords:** diet, survival, heart disease, vegetables, nutrients

## Abstract

Few studies have assessed the association between potato consumption and mortality, especially cardiovascular disease (CVD) mortality. Our objective was to investigate the association between consumption of boiled potatoes and all-cause and CVD mortality in a Norwegian population. We used data from the population based HUNT3 study in Norway, with data on boiled potato consumption frequency in 2006–2008 from 49,926 males and females aged 20 years or above. All-cause and CVD mortality were identified during 10 years follow-up through the national Cause of Death Registry, which is virtually complete. We used Cox regression to estimate hazard ratio (HR) with a 95% confidence interval (CI) for death controlling for potential confounders, and conducted additional analyses stratified by sex, body mass index (BMI) ±25 kg/m^2^, and age ±65 years. There were 4,084 deaths and 1,284 of these were due to CVD. Frequency of boiled potato consumption was not associated with all-cause mortality, nor with CVD mortality. Compared to those individuals who consumed boiled potatoes less than once weekly, those who reported to consume boiled potatoes 1–3 times per week had an adjusted HR (95% CI) of 1.12 (0.89, 1.41) for all-cause mortality and 1.20 (0.78, 1.86) for CVD mortality. Individuals who consumed boiled potatoes 4–6 times per week had HRs of 0.97 (0.78, 1.21) and 1.03 (0.68, 1.55), for all-cause and CVD mortality, respectively, whereas those who consumed boiled potatoes more than once daily had HRs of 1.04 (0.83, 1.29) and 1.09 (0.73, 1.63) for all-cause and CVD mortality, respectively. There was no evidence of differential associations for males vs. females, nor between people with BMI ± 25 kg/m^2^. The associations between frequency of boiled potato consumption and all-cause mortality showed different patterns between those younger vs. older than 65 years, with a tendency of increased risk only in the oldest age group. In conclusion, frequency of consumption of boiled potatoes was not associated with all-cause or CVD mortality in the HUNT population in Norway.

## Introduction

Europe has the highest level of potato consumption in the world, with almost 90 kg per capita per year (1). Potatoes contain several key nutrients, including potassium, dietary fiber, and vitamin C. On the other hand, potatoes have high carbohydrate content and are considered to be a high glycaemic index food, making the nutritional value of potatoes uncertain (2). Some prospective observational studies have reported positive associations between potato consumption and incidence of type 2 diabetes (3, 4) and hypertension (5), whereas others have reported no such associations (6, 7). Two recent systematic reviews and dose-response meta-analyses of prospective cohort studies found no associations between total potato consumption and risk of all-cause mortality (8, 9). However, frequent consumption of fried potatoes was associated with all-cause mortality in a North American cohort (10). So far, there have been too few studies to perform meta-analyses about the association between potato intake and CVD mortality. Pietinen et al. (11) reported an inverse association between intake of potatoes and coronary heart disease mortality in a cohort of 21,930 middle-aged, smoking Finnish males. Contrary, another study in 69,313 Swedish males and females, reported no associations between potato consumption and CVD mortality (6). Data from the National Health and Nutrition Examination Surveys (NHANES) 1999–2010 showed an association between total potato consumption and all-cause and CVD mortality in crude analyses, but the association was largely attenuated after adjusting for hypertension and diabetes (12). These inconsistent findings may be due to different preparation methods of potatoes used in the different cohorts and available evidence is insufficient to reach a conclusion about the association between potato consumption and mortality. There are some data showing that individuals with a high potato consumption have a less favorable profile of metabolic and cardiovascular risk factors, such as circulating lipids, abdominal obesity, blood pressure profiles, and the metabolic syndrome (12, 13). The aim of this study was to investigate the associations between the frequency of consumption of boiled potatoes and risk of all-cause and CVD mortality in a Norwegian population.

## Subjects and Methods

### Study Population

**Figure 1:**
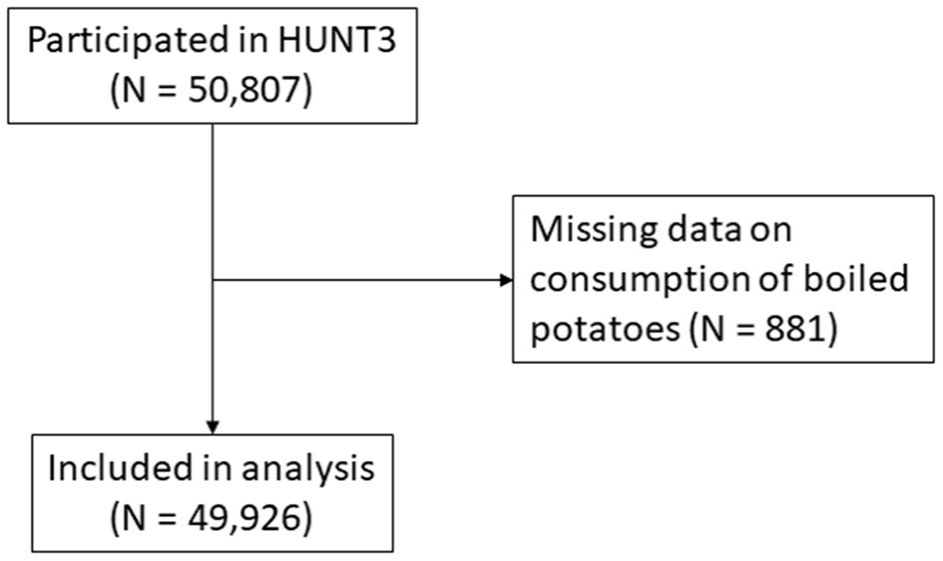
Flow diagram of participants included in the analysis.

We included participants from the HUNT3 Study, undertaken in 2006–2008. The HUNT Study is a longitudinal population-based health study among residents who are 20 years or older in one county in Norway and 50,807 participated in HUNT3. Detailed information about the HUNT-studies and cohort profile is available at http://www.ntnu.edu/hunt. We included participants with valid data on potato consumption (*n* = 49,926) in this study (Figure 1). All participants provided informed, written consent. The study was approved by the Regional Committee for Ethics in Medical Research (no. 2017/1503 REK midt).

### Assessment of Potato Consumption and Possible Confounders

The participants completed questionnaires about their habitual intake of various food groups, including vegetables, fruits, fish, meat, pasta, and rice. Consumption of boiled potatoes was assessed by the questions “How often do you normally eat boiled potatoes?” and this question had four response alternatives; (1) <once per week, (2) 1–3 times per week, (3), 4–6 times per week, and (4) at least once daily.

The HUNT3 study includes survey questionnaires on a range of other health and lifestyle-related topics and a clinical examination of standardized measures of height, body weight and blood pressure. Height and body weight were measured with the participants wearing light clothes (without shoes). Height was measured to the nearest centimetre and weight to the nearest half kilogram. Body mass index (BMI) was calculated as kg/m^2^. CVD, diabetes, and cancer at baseline were self-reported. Blood pressure was measured three times using a Dinamap 845XT (Critikon), and we used the mean of the second and third measurements in the analyses. We categorised those with systolic blood pressure ≥140 mmHg and/or diastolic blood pressure ≥90 mmHg and/or currently taking blood pressure medication as having hypertension. Participants also reported their smoking status, leisure time physical activity, occupational physical activity, alcohol consumption, and consumption of vegetables, high-fat fish, pasta/rice, and sausages/hamburgers in detailed questionnaires.

### Follow-Up

We used the unique personal identification number of all Norwegian citizens to link data from HUNT to information from the national Cause of Death Registry on primary cause of death classified according to the International Classification of Disease (ICD). Mortality from all causes and from CVD (i.e., ICD-10: I00-I99) were recorded from baseline in 2006-2008 until the date of death or end of follow-up 31.12.2016, whichever occurred first.

### Statistical Analyses

We used Cox regression to estimate hazard ratios (HRs) with 95% confidence intervals (CIs) of death from all causes and from CVD within each category of boiled potato consumption, compared to the reference category of individuals who reported to eat potatoes less than once weekly. All estimates were adjusted for age and sex, as well as for CVD at baseline (yes or no), cancer at baseline (yes or no), hypertension at baseline (yes or no), BMI (<18.5, 18.5–24.9, 25.0–29.9, or ≥30.0 kg/m^2^), leisure time physical activity (none, <1, 1, 2–3, or ≥4 times per week), occupational physical activity (mostly sitting, much walking, much walking and lifting, heavy physical work, or unknown/not employed), smoking (never, former, current, or unknown), and habitual intake of alcohol (≤1 per month, ≤1 per week, ≥2 per week, or abstainer), vegetables (≤3 times per week, 4–6 times per week, ≥1 times per day), high-fat fish (≤3 times per month, 1–3 times per week, ≥4 times per week), pasta/rice (≤3 times per month, 1–3 times per week, ≥4 times per week), and sausages/hamburgers (≤3 times per month, 1–3 times per week, ≥4 times per week). The proportional hazard assumption was assessed by tests of Schoenfeld residuals and inspection of log-log plots. Additional analyses were stratified by sex (male, female), by BMI (±25 kg/m^2^) and by age (±65 years). We also conducted three sensitivity analyses: (1) (we did not adjust for intake of pasta/rice) since this may be a mediator of the association under study, (2) we excluded participants with CVD at baseline to reduce possible confounding by ill health, and for the same reason, we (3) excluded participants who died within the first 2 years of follow-up. Statistical tests were two-sided, and all analyses were performed using Stata for Windows (Version 15 © StataCorp LP, 1985-2017).

## Results

Table 1 shows the baseline characteristics of the participants according to boiled potato consumption. About two thirds of the participants reported to eat boiled potatoes four times or more per week. Compared with those who had a low intake of boiled potatoes, those with a high consumption were older, had more often a sedentary work type, were less physically active, had higher prevalence of hypertension and CVD, and had a lower intake of pasta/rice.

**Table 1:** Selected participant characteristics according to boiled potato consumption.

	**Boiled potato consumption per week**			
**Variable**	**<1**	**1–3**	**4–6**	**Daily**
Number of participants	2,712	12,808	19,942	14,464
Age, years, mean (SD)	38.8 (15.5)	43.9 (13.8)	53.4 (14.2)	63.3 (13.3)
Women/men	1,633/1,079	7,354/5,454	10,583/9,359	7,677/6,787
Body mass index, kg/m^2^	26.4 (4.7)	26.7 (4.4)	27.3 (4.3)	27.6 (4.4)
Cigarette smoking, current smoker	27%	25%	23%	25%
Alcohol consumption, >1/week	13%	16%	17%	12%
Work type, sedentary	31%	32%	25%	14%
Physical activity, <1/week	23%	21%	21%	24%
Hypertension	21%	24%	40%	57%
Cardiovascular disease	4%	4%	8%	15%
Pasta/rice consumption, ≥4/week	23%	9%	4%	3%

A total of 4,084 deaths occurred with 1,284 from CVD over 10 years of follow-up. There were no associations between intake of boiled potatoes and all-cause mortality or CVD mortality (Table 2). The same was true also when the analyses were stratified by sex (Table 3) and by BMI (Table 4). In the analyses stratified for BMI ± 25 kg/m^2^, the HRs suggest lower CVD mortality in those who had a healthy BMI and who consumed boiled potatoes once per week or more often compared to the reference group, whereas the opposite was evident for those with overweight/obesity (Table 4). When the analyses were stratified for age ±65 years, we observed a tendency of increased all-cause mortality associated with frequent consumption of boiled potatoes among those in the oldest age group, whereas for those below 65 years the HR was highest for those in the reference group (<once weekly consumption) (Table 5).

**Table 2:** Boiled potato consumption in relation to mortality from all causes and from cardiovascular diseases (CVD).

**Frequency of boiled potato consumption**	**No. of persons years**	**No. of cases**	**Crude HR**	**Adjusted HR^a^**	**95% CI^a^**
**All-Cause Mortality**					
<1 per week	257,933	90	1.00	1.00	Reference
1–3 per week	1,220,518	419	0.98	1.12	0.89–1.41
4–6 per week	1,889,300	1,328	0.83	0.97	0.78–1.21
≥1 per day	1,321,989	2,247	0.90	1.04	0.83–1.29
**CVD Mortality**					
<1 per week	257,933	26	1.00	1.00	Reference
1–3 per week	1,220,518	109	0.97	1.20	0.78–1.86
4–6 per week	1,889,300	402	0.85	1.03	0.68–1.55
≥1 per day	1,321,989	747	0.93	1.09	0.73–1.63

a *Adjusted for age (time scale), sex (woman, man), CVD at baseline (no, yes), diabetes at baseline (no, yes), hypertension at baseline (no, yes), cancer at baseline (no, yes), body mass index (<18.5, 18.5–24.9, 25.0–29.9, ≥30.0 kg/m^2^), work type (sitting, waling, walking and lifting, heavy work, unknown/not employed), frequency of physical activity (none, <1, 1, 2–3, ≥4 times per week), smoking (never, former, current, unknown), alcohol past year (≤1 month, 1–3 per month, ≥1 per week, never), and intake of vegetables (≤3 times per week, 4–6 times per week, ≥1 times per day), high-fat fish (≤3 times per month, 1–3 times per week, ≥4 times per week), pasta/rice (≤3 times per month, 1–3 times per week, ≥4 times per week), and sausages/hamburgers (≤3 times per month, 1–3 times per week, ≥4 times per week)*.

**Table 3:** Boiled potato consumption in relation to mortality from all causes and from cardiovascular diseases (CVD), stratified by sex.

	**Males**					**Females**				
**Frequency of boiled potato consumption**	**No. of person years**	**No. of cases**	**Crude HR**	**Adjusted HR^a^**	**95% CI^a^**	**No. of person years**	**No. of cases**	**Crude HR**	**Adjusted HR^a^**	**95% CI^a^**
**All-Cause Mortality**										
<1 per week	102,013	46	1.00	1.00	Reference	155,920	44	1.00	1.00	Reference
1–3 per week	519,144	200	0.84	0.98	0.71-1.35	701,374	219	1.14	1.29	0.92–1.79
4–6 per week	880,403	747	0.71	0.85	0.62-1.15	1,008,897	581	0.96	1.10	0.80–1.52
≥1 per day	611,966	1,214	0.77	0.88	0.65-1.20	710,022	1,033	1.05	1.21	0.88–1.65
**CVD Mortality**										
<1 per week	102,013	14	1.00	1.00	Reference	155,920	12	1.00	1.00	Reference
1–3 per week	519,144	54	0.77	1.02	0.56-1.86	701,374	55	1.23	1.44	0.76–2.74
4–6 per week	880,403	233	0.67	0.90	0.51-1.57	1,008,897	169	1.06	1.15	0.63–2.11
≥1 per day	611,966	415	0.77	0.95	0.55-1.65	710,022	332	1.12	1.23	0.67–2.24

a *Adjusted for age (time scale), CVD at baseline (no, yes), diabetes at baseline (no, yes), hypertension at baseline (no, yes), cancer at baseline (no, yes), body mass index (<18.5, 18.5–24.9, 25.0–29.9, ≥ 30.0 kg/m^2^), work type (sitting, walking, walking and lifting, heavy work, unknown/not employed), frequency of physical activity (none, <1, 1, 2–3, ≥4 times per week), smoking (never, former, current, unknown), alcohol past year (≤1 month, 1–3 per month, ≥1 per week, never), and intake of vegetables (≤3 times per week, 4–6 times per week, ≥1 times per day), high-fat fish (≤3 times per month, 1–3 times per week, ≥4 times per week), pasta/rice (≤3 times per month, 1–3 times per week, ≥4 times per week), and sausages/hamburgers (≤3 times per month, 1–3 times per week, ≥4 times per week)*.

**Table 4:** Boiled potato consumption in relation to mortality from all causes and from cardiovascular diseases (CVD), stratified by body mass index (BMI) ±25 kg/m^2^.

	**<25 kg/m^2^**					**≥25 kg/m^2^**				
**Frequency of boiled potato consumption**	**No. of person years**	**No. of cases**	**Crude HR**	**Adjusted HR^a^**	**95% CI^a^**	**No. of person years**	**No. of cases**	**Crude HR**	**Adjusted HR^a^**	**95% CI^a^**
**All-Cause Mortality**										
<1 per week	111,966	31	1.00	1.00	Reference	144,374	56	1.00	1.00	Reference
1–3 per week	461,224	110	0.73	0.92	0.61–1.39	751,762	299	1.13	1.26	0.94–1.69
4–6 per week	584,260	407	0.73	0.85	0.58–1.25	1,294,031	880	0.95	1.08	0.82–1.43
≥1 per day	365,624	674	0.75	0.87	0.59–1.27	946,898	1,496	1.06	1.15	0.87–1.51
**CVD Mortality**										
<1 per week	111,966	11	1.00	1.00	Reference	144,374	14	1.00	1.00	Reference
1–3 per week	461,224	28	0.65	0.92	0.44–1.95	751,762	77	1.25	1.60	0.89–2.87
4–6 per week	584,260	108	0.53	0.64	0.33–1.27	1,294,031	279	1.21	1.50	0.86–2.61
≥1 per day	365,624	229	0.62	0.75	0.39–1.46	946,898	491	1.27	1.50	0.87–2.61

a *Adjusted for age (time scale), sex (woman, man), CVD at baseline (no, yes), diabetes at baseline (no, yes), hypertension at baseline (no, yes), cancer at baseline (no, yes),work type (sitting, walking, walking and lifting, heavy work, unknown/not employed), frequency of physical activity (none, <1, 1, 2–3, ≥4 times per week), smoking (never, former, current, unknown), alcohol past year (≤1 month, 1–3 per month, ≥1 per week, never), and intake of vegetables (≤3 times per week, 4–6 times per week, ≥1 times per day), high-fat fish (≤3 times per month, 1–3 times per week, ≥4 times per week), pasta/rice (≤3 times per month, 1–3 times per week, ≥4 times per week), and sausages/hamburgers (≤3 times per month, 1–3 times per week, ≥4 times per week)*.

**Table 5:** Boiled potato consumption in relation to mortality from all causes and from cardiovascular diseases (CVD), stratified by age ±65 years.

	**<65 years**					**≥65 years**				
**Frequency of boiled potato consumption**	**No. of person years**	**No. of cases**	**Crude HR**	**Adjusted HR^a^**	**95% CI^a^**	**No. of person years**	**No. of cases**	**Crude HR**	**Adjusted HR^a^**	**95% CI^a^**
**All-cause mortality**										
<1 per week	243,270	39	1.00	1.00	Reference	14,663	56	1.00	1.00	Reference
1–3 per week	1,145,140	181	0.76	0.82	0.57–1.16	75,379	299	1.16	1.36	1.00–1.85
4–6 per week	1,525,805	344	0.67	0.74	0.53–1.04	363,495	880	1.02	1.16	0.87–1.55
≥1 per day	737,430	279	0.75	0.71	0.50–1.01	584,559	1,496	1.10	1.24	0.93–1.64
**CVD mortality**										
<1 per week	243,270	11	1.00	1.00	Reference	14,663	14	1.00	1.00	Reference
1–3 per week	1,145,140	28	0.64	0.67	0.27–1.66	75,379	77	1.13	1.41	0.86–2.31
4–6 per week	1,525,805	108	0.83	0.86	0.36–2.03	363,495	279	0.94	1.07	0.67–1.70
≥1 per day	737,430	229	0.90	0.78	0.32–1.87	584,559	491	1.03	1.14	0.72–1.80

a *Adjusted for age (time scale), sex (woman, man), CVD at baseline (no, yes), diabetes at baseline (no, yes), hypertension at baseline (no, yes), cancer at baseline (no, yes),work type (sitting, walking, walking and lifting, heavy work, unknown/not employed), frequency of physical activity (none, <1, 1, 2–3, ≥4 times per week), smoking (never, former, current, unknown), alcohol past year (≤1 month, 1–3 per month, ≥1 per week, never), and intake of vegetables (≤3 times per week, 4–6 times per week, ≥1 times per day), high-fat fish (≤3 times per month, 1–3 times per week, ≥4 times per week), pasta/rice (≤3 times per month, 1–3 times per week, ≥4 times per week), and sausages/hamburgers (≤3 times per month, 1–3 times per week, ≥4 times per week)*.

### Sensitivity Analyses

We observed only minor changes to the estimates when we repeated the analyses without adjusting for intake of pasta/rice (Supplementary Table 1). Neither did excluding individuals with CVD at baseline (Supplementary Table 2) nor excluding individuals who died during the first 2 years of follow-up (Supplementary Table 3) change the estimates substantially.

## Discussion

We examined the association between the frequency of boiled potato consumption and all-cause and CVD mortality in a Norwegian population. Boiled potatoes are a principal element in the traditional diet for this population, with two thirds of the participants reporting to eat boiled potatoes four times or more per week. We observed no clear associations between intake of boiled potatoes and mortality, neither from all causes nor from CVD. The associations were similar in males and females. Among individuals with BMI <25 kg/m^2^, the HRs were lower for those with frequent consumption of boiled potatoes, whereas there was an opposite trend for those with BMI ≥25 kg/m^2^. Opposite associations between frequency of potato consumption and mortality were also evident in those who were below and above 65 years of age. We are unsure how to interpret these differing associations for BMI categories and for age, and it is worth noticing that for neither stratification was there any clear dose-response between frequency of consumption and mortality.

Previous studies on the associations between consumption of potatoes and risk of type 2 diabetes, hypertension, and CVD, as well as CVD and all-cause mortality have shown conflicting results. Some prospective observational studies report that potato consumption is positively associated with the risk of type 2 diabetes and hypertension (3–5, 14). One of these studies is from the China Health and Nutrition Survey (CHNS) (14). However, in the same population, researchers found that low and moderate, but not high, levels of total potato consumption were inversely associated with all-cause mortality (15). We previously reported some weak positive associations between boiled potato consumption and several CVD risk factors (high waist circumference, high circulating triglycerides, and prevalence of the metabolic syndrome) in a cross-sectional investigation of the HUNT population (13). Here we observe that these associations do not transfer to increased mortality in this population. Our data are also consistent with findings from two prospective cohort studies in Swedish adults, who also have a high consumption of boiled potatoes (6). Larsson and Wolk (6) observed no evidence that consumption of boiled potatoes associated with the risk of major CVD events or mortality form CVD.

Different preparation methods of potatoes used in the different cohorts may partly explain the diverse findings. In our study, we investigated boiled potato consumption, which is assumed to be the healthiest preparation method. Roasted and fried potatoes carry more salt and fats, with acrylamide formation as an additional problem if the potatoes are prepared at temperatures above 120°C (16). The unfavorable cardiometabolic effects of potatoes are generally attributed to their high content of starch, leading to an exceptionally high glycaemic index (17). The glycaemic index of potatoes is, however, dependent on the preparation method, with boiled potatoes eliciting higher glycaemic response than french fries (18). Foods with high glycaemic index induce a rapid rise in blood glucose (19) and such foods are suggested to cause weight gain by redirecting nutrition from oxidation in insulin-sensitive tissue to storage as fat (20). Postprandial hyperglycaemia is an important risk factor for incident CVD events and this relationship extends below the diabetic threshold (21). However, there were no indications for an increased CVD mortality with high consumption of boiled potatoes in this Norwegian population. Potatoes are typically part of a complex meal (2), together with other foods with lower glycaemic index and which reduces the overall postprandial glucose response of the meal (22).

The strengths of this study are the population-based nature of the data, the prospective design, detailed information on lifestyle and health-related factors, and the completeness of follow-up by linkage with the population-based Norwegian Cause of Death Registry. There are also some limitations to our study. The diet questionnaire in HUNT did not capture information about the portion sizes, so we were not able to calculate the total consumption of potatoes, only the frequency of intake. Neither could we calculate the total energy intake or the distribution of macronutrients in the diet of the participants based on the questionnaire, and the lack of these data represents a main limitation. Additionally, we had no information about consumption of potatoes prepared by different cooking methods, as for example frying. It is likely that the frequency of boiled potato consumption could be inversely correlated with intake of potatoes prepared otherwise. Finally, we cannot rule out residual confounding due to poorly or unmeasured lifestyle and health related factors or confounding by ill health that causes both dietary changes and increase mortality.

In conclusion, the frequency of boiled potato consumption was not associated with all-cause or CVD mortality in this population.

## Data Availability Statement

Data described in the article, code book, and analytic code will not be made available because the Trøndelag Health Study (HUNT) does not allow for such sharing. The data are stored in HUNT databank and biological material in HUNT biobank. HUNT Research Centre has permission from the Norwegian Data Inspectorate to store and handle these data. The key identification in the data base is the personal identification number given to all Norwegians at birth or immigration, whilst de-identified data are sent to researchers upon approval of a research protocol by the Regional Ethical Committee and HUNT Research Centre. To protect participants' privacy, HUNT Research Centre aims to limit storage of data outside HUNT databank, and cannot deposit data in open repositories. HUNT databank has precise information on all data exported to different projects and are able to reproduce these on request. There are no restrictions regarding data export given approval of applications to HUNT Research Centre. For more information see: http://www.ntnu.edu/hunt/data.

## Ethics Statement

The studies involving human participants were reviewed and approved by the Regional Committee for Ethics in Medical Research, Central Norway. The patients/participants provided their written informed consent to participate in this study.

## Author Contributions

TM designed research (project conception, development of overall research plan, and study oversight) and had primary responsibility for final content. TM and TN conducted research and wrote the manuscript. TN analyzed data and performed statistical analyses. All authors have read and approved the final manuscript.

## Conflict of Interest

The authors declare that the research was conducted in the absence of any commercial or financial relationships that could be construed as a potential conflict of interest.
