# Supplementary material for: Frequency of Boiled Potato Consumption and All-Cause and Cardiovascular Disease Mortality in the Prospective Population-Based HUNT Study
Source: Front Nutr. 2021 Jul 19;8:681365. doi: 10.3389/fnut.2021.681365 (PMC8326454; doi:10.3389/fnut.2021.681365)
Supplement: Supplementary file 1 [file Table_1.docx]

**Supplementary Table 1. Boiled potato consumption in relation to mortality from all causes and from cardiovascular diseases (CVD), without adjusting for intake of pasta/rice.**

| Frequency of boiled  potato consumption | No. of  persons years | No. of  cases | Crude HR | Adjusted HR^1^ | 95% CI^1^ |
| --- | --- | --- | --- | --- | --- |
| All-cause mortality |  |  |  |  |  |
| <1 per week | 25,7933 | 90 | 1.00 | 1.00 | Reference |
| 1-3 per week | 122,0518 | 419 | 0.98 | 1.10 | 0.87-1.39 |
| 4-6 per week | 188,9300 | 1328 | 0.83 | 0.96 | 0.77-1.20 |
| ≥1 per day | 132,1989 | 2247 | 0.90 | 1.04 | 0.83-1.29 |
| CVD mortality |  |  |  |  |  |
| <1 per week | 25,7933 | 26 | 1.00 | 1.00 | Reference |
| 1-3 per week | 122,0518 | 109 | 0.97 | 1.18 | 0.76-1.82 |
| 4-6 per week | 188,9300 | 402 | 0.85 | 1.02 | 0.68-1.53 |
| ≥1 per day | 132,1989 | 747 | 0.93 | 1.09 | 0.73-1.64 |

^1^Adjusted for age (time scale), sex (woman, man), CVD at baseline (no, yes), diabetes at baseline (no, yes), hypertension at baseline (no, yes), cancer at baseline (no, yes), body mass index (<18.5, 18.5-24.9, 25.0-29.9, ≥ 30.0 kg/m^2^), work type (sitting, walking, walking and lifting, heavy work, unknown/not employed), frequency of physical activity (none, <1, 1, 2-3, ≥4 times per week), smoking (never, former, current, unknown), alcohol past year (≤1 month, 1-3 per month, ≥1 per week, never), and intake of vegetables (≤3 times per week, 4-6 times per week, ≥1 times per day), high-fat fish (≤3 times per month, 1-3 times per week, ≥4 times per week) , and sausages/hamburgers (≤3 times per month, 1-3 times per week, ≥4 times per week).

**Supplementary Table 2. Boiled potato consumption in relation to mortality from all causes and from cardiovascular diseases (CVD), without individuals with CVD at baseline**

| Frequency of boiled  potato consumption | No. of  persons years | No. of  cases | Crude HR | Adjusted HR^1^ | 95% CI^1^ |
| --- | --- | --- | --- | --- | --- |
| All-cause mortality |  |  |  |  |  |
| <1 per week | 24,9170 | 65 | 1.00 | 1.00 | Reference |
| 1-3 per week | 118,0108 | 314 | 0.94 | 1.08 | 0.82-1.42 |
| 4-6 per week | 175,6920 | 926 | 0.86 | 0.99 | 0.76-1.28 |
| ≥1 per day | 114,1778 | 1459 | 0.93 | 1.05 | 0.81-1.35 |
| CVD mortality |  |  |  |  |  |
| <1 per week | 24,9170 | 14 | 1.00 | 1.00 | Reference |
| 1-3 per week | 118,0108 | 62 | 0.97 | 1.20 | 0.66-2.17 |
| 4-6 per week | 175,6920 | 228 | 0.99 | 1.17 | 0.67-2.04 |
| ≥1 per day | 114,1778 | 416 | 1.12 | 1.32 | 0.76-2.30 |

^1^Adjusted for age (time scale), sex (woman, man), diabetes at baseline (no, yes), hypertension at baseline (no, yes), cancer at baseline (no, yes), body mass index (<18.5, 18.5-24.9, 25.0-29.9, ≥ 30.0 kg/m^2^), work type (sitting, walking, walking and lifting, heavy work, unknown/not employed), frequency of physical activity (none, <1, 1, 2-3, ≥4 times per week), smoking (never, former, current, unknown), alcohol past year (≤1 month, 1-3 per month, ≥1 per week, never), and intake of vegetables (≤3 times per week, 4-6 times per week, ≥1 times per day), high-fat fish (≤3 times per month, 1-3 times per week, ≥4 times per week), pasta/rice (≤3 times per month, 1-3 times per week, ≥4 times per week), and sausages/hamburgers (≤3 times per month, 1-3 times per week, ≥4 times per week).

**Supplementary Table 3. Boiled potato consumption in relation to mortality from all causes and from cardiovascular diseases (CVD), without individuals who died the first two years of follow-up**

| Frequency of boiled  potato consumption | No. of  persons years | No. of  cases | Crude HR | Adjusted HR^1^ | 95% CI^1^ |
| --- | --- | --- | --- | --- | --- |
| All-cause mortality |  |  |  |  |  |
| <1 per week | 20,3811 | 77 | 1.00 | 1.00 | Reference |
| 1-3 per week | 96,4719 | 374 | 1.02 | 1.17 | 0.91-1.50 |
| 4-6 per week | 149,1320 | 1211 | 0.87 | 1.04 | 0.82-1.31 |
| ≥1 per day | 103,4417 | 2017 | 0.95 | 1.10 | 0.87-1.39 |
| CVD mortality |  |  |  |  |  |
| <1 per week | 20,3811 | 22 | 1.00 | 1.00 | Reference |
| 1-3 per week | 96,4719 | 96 | 1.01 | 1.28 | 0.80-2.05 |
| 4-6 per week | 149,1320 | 362 | 0.90 | 1.11 | 0.71-1.73 |
| ≥1 per day | 103,4417 | 654 | 0.96 | 1.15 | 0.74-1.78 |

^1^Adjusted for age (time scale), sex (woman, man), CVD at baseline (no, yes), diabetes at baseline (no, yes), hypertension at baseline (no, yes), cancer at baseline (no, yes), body mass index (<18.5, 18.5-24.9, 25.0-29.9, ≥ 30.0 kg/m^2^), work type (sitting, walking, walking and lifting, heavy work, unknown/not employed), frequency of physical activity (none, <1, 1, 2-3, ≥4 times per week), smoking (never, former, current, unknown), alcohol past year (≤1 month, 1-3 per month, ≥1 per week, never), and intake of vegetables (≤3 times per week, 4-6 times per week, ≥1 times per day), high-fat fish (≤3 times per month, 1-3 times per week, ≥4 times per week), pasta/rice (≤3 times per month, 1-3 times per week, ≥4 times per week), and sausages/hamburgers (≤3 times per month, 1-3 times per week, ≥4 times per week.
